# Meetings of Societies

**Published:** 1903-12

**Authors:** 


### MEETINGS OF SOCIETIES.

---

### Bristol Medico-Chirurgical Society.

*Annual Meeting, October 14th, 1903.*

After a vote of thanks to the retiring President (Mr. G. Munro Smith) had been unanimously passed, Mr. Paul Bush took the chair, and gave an introductory address [see p. 289]. Subsequently a cordial vote of thanks was given to Mr. Bush for his able address.

Mr. Bush, as retiring secretary, read the Secretary's Annual Report. The balance in hand was £153 19s. 5d., as compared with £80 2s. 6d. at the end of the previous session. The Society had to regret the loss of four members through death, viz.: Dr. J. G. Swayne, who was president in the session of 1880—1881, Mr. W. M. Barclay, Mr. H. T. Rudge, and Dr. W. A. Perry. The report was adopted, and thanks given to the secretary for his services.

The Editorial Secretary (Mr. James Taylor), and the Honorary Librarian of the Medical Library (Mr. C. K. Rudge), then read their Annual Reports, which were adopted.

The following officers were chosen for the ensuing year:— President-elect, Dr. Reginald Eager; Hon. Secretary, Mr. H. F.

Mole; Members of Committee, Dr. J. Michell Clarke, Mr. J. Dacre, Dr. B. M. H. Rogers, Mr. J. Taylor, Dr. H. Waldo, and Dr. P. Watson Williams; and Members of the Library Committee, Mr. C. K. Rudge, Mr. G. Munro Smith, and Dr. A. Ogilvy.

---

*November 11th, 1903.*

Mr. J. Paul Bush, C.M.G., President, in the Chair.

Mr. T. Carwardine showed (1) a small **ovarian dermoid containing mammæ.** A well-formed nipple and areola were visible at one place, and above this another small swelling of a similar character. The patient was pregnant at the time of operation, and the pedicle of the tumour was twisted. (2) **A prostate gland which had been removed by the supra-pubic route.** The patient had made a good recovery, and now had complete control of the bladder. For comparison with this, prostatic adenomata removed by McGill's method were also shown.—Mr. C. A. Morton alluded to an operation of the kind he had performed in an old man, who also had a vesical calculus. There had been a good deal of hemorrhage, but the shock had not been great. Recovery followed.—Mr. Hey Groves, referring to the ovarian dermoid, asked how far pregnancy had advanced at the time of operation. It had been observed that dermoid cysts were specially liable to torsion of their pedicles, and also that pregnancy seemed to favour such torsion in all ovarian tumours.—Dr. Watson Williams referred to the tendency of patients to develop insanity after prostatic operations.—Mr. Roger Williams discussed the origin of mammæ in dermoids. According to the sequestration theory, ovarian dermoids arose through the proximity of the ovaries to the ectoderm in the early stages of development. This permitted the sequestration of ectoderm cells in the ovaries. There had been a tendency to deny that these prominences in ovarian dermoids were really nipples. They might contain sebaceous and other types of glandular tissue. The teeth in dermoids might be similarly explained by the sequestrations of ectoderm cells.—Mr. Carwardine stated that the patient with the dermoid had been three months pregnant. There had been many cases in which insanity had developed after prostatectomy, but probably not in so great a proportion as after the operation of double orchidtectomy. If there was a strong history of insanity in a patient, or if a patient had already shown a tendency to insanity, the operation was exceptionally risky. But any operation on the genito-urinary organs in old people might lead to insanity.

Dr. J. Odery Symes showed **microscopical specimens of trypanosomes in human blood.**—[For his remarks on this parasite *vide* p. 325.]—Dr. Markham Skerritt, under whose care the patient had come, after previously being under the care of Sir Patrick Manson and others, read notes of the previous history of the patient. Details of the case have been published in the *British Medical Journal* of May 30th and (of the necropsy) Dec. 5th, p. 1462.—Dr. EDGEWORTH asked if the hypnococcus had been found, and Dr. SYMES replied in the negative.

Mr. PRICHARD read a paper on **the subsequent history of a case of pylorectomy.** The history of the case up to the time when pylorectomy had been performed, and the description of the operation and its immediate results, had been given to the Society on a previous occasion.* For about twelve months after the operation the patient had remained well, and had had good digestion and gained flesh. In September of this year she began to have pain on the right side of the abdomen with tenderness there, and constipation. The temperature rose to 102°, and dulness in the appendix region developed. Laparotomy was performed and pus evacuated from behind the uterus. Three days later the patient died from peritonitis. A *post-mortem* examination showed new growth entensively involving both ovaries, and infiltration and thickening of the wall of the cæcum and the smaller curvature of the stomach. The appendix was normal. The nature of the growth in the pylorus at the time when pylorectomy was performed had been doubtful; some of those who saw the tumour had regarded it as an inflammatory growth, others as malignant disease (sarcoma). All the diseased parts were shown by Mr. Prichard.—Mr. MORTON still thought the original disease of the pylorus had been inflammatory, and that the cause of death had been a primary sarcoma of the ovaries with secondary growth in the cæcum and smaller curvature of the stomach. He alluded to the great difficulty there was in distinguishing between inflammatory exudation and sarcoma microscopically.—Mr. MUNRO SMITH thought the sections showed that the growth in the ovaries and pylorus was sarcomatous.

Dr. MICHELL CLARKE read notes on the following cases, and showed specimens and microscopical preparations obtained from them. (1) Gumma of the spinal cord from acquired syphilis in a man aged 48. The gumma was growing from the meninges into the cord at about the position of the pyramidal tract in the lumbar region, and the cord was much destroyed. The symptoms included localised atrophy of the muscles of the left thigh and calf, girdle pain at the level of the 10th to 12th dorsal roots, rigidity and increase of deep reflexes. In addition, there was a large gumma of the brain in the region of the right arm and leg centres. (2) Meningomyelitis of the cord from a case of hereditary syphilis. The specimens showed meningomyelitis with well-marked endarteritis of branches of the anterior spinal artery, degeneration in the column of Goll above, and in the crossed pyramidal tracts below, the seat of

* Vide *Bristol M. Chir. Journal*, 1902, xx. 285.

the chief lesion, which was at the level of the 3rd and 4th dorsal roots. The meningitis, which constituted an internal pachymeningitis, had a considerable vertical extent over the dorsal and lumbar regions of the cord. The patient was a youth of 21, who a year previously had been treated for cerebral syphilis. He showed old interstitial keratitis and Hutchinson's teeth. The cord lesions caused complete paraplegia, with incontinence of urine and fæces, exaggeration of deep reflexes, and loss of all forms of sensation below the 4th dorsal roots. The upper limit of the lesion was clinically so definitely localised that laminectomy was done, as there seemed a prospect of relieving him by operation. The operation was performed by Mr. Lacy Firth, and was well borne by the patient, but afforded no relief to the symptoms. (3) Sections of the spinal cord showing a central cavity in the cervical region (hydromyelia). This was found accidentally in a child of three who died from a tumour (glioma) of the cerebellum. There were no symptoms of the cavity during life. Specimens were also shown indicating the presence of a descending tract in the anterolateral region of the cord (? from Deiter's nucleus), and of the extreme compression of the bulb which can be produced by a slowly growing tumour without causing degenerations.

Dr. Walter Swayne read a paper on a case of **Cæsarian section.** The obstruction to parturition was a tumour in the pelvis. The patient had been admitted to the Bristol Royal Infirmary for abdominal pains which had been thought to be labour pains, but which had ceased after an enema had been given. A large hard tumour occupied the pelvis, only leaving room for the passage of two fingers side by side between its anterior surface and the symphysis pubis. It was doubtful whether the tumour was a fibroid or an ovarian cyst. The period of pregnancy was a little doubtful, for, although the fundus of the uterus reached the ensiform cartilage, this was partly due to the fact that the fœtal head could not descend into the pelvis. It was decided to wait until full term before operating. [We hope to publish Dr. Swayne's paper in full in our next issue.]

Mr. Roger Williams read a paper on **subungual exostosis.** This growth he had found affecting the great toe in thirty-four out of thirty-five collected cases. The little toe was the next most frequently affected. The tumour was attached to the tibial side of the ungual phalanx of the great toe. The cortex of the tumour was continuous with that of the phalanx, and the central part with the spongy bone of the interior of the phalanx. Sometimes the exostosis was attached to the external or dorsal surface of the phalanx. In the growing stage, fibro-cartilage lay between it and the ungual phalanx, and also covered its outer surface. The growth was innocent, and originated from a cartilaginous rest beneath the periosteum. It was surprising to find it stated in a recently-issued book that these growths were often sarcomatous, and the statement was wrong. Attention was usually first attracted to the growths in adolescence. The majority arose spontaneously. In ten out of thirteen of his cases no cause could be found. The growth had no connection with the cartilage of the epiphysis. When there were multiple exostoses on the toes and in other parts, the subungual exostosis was not found in association with them. Rarely the growth was found affecting a finger. The speaker thought the evidence was in favour of the view that the exostosis was the representative of a lost *preaxial* hallux. In removing these tumours it was advisable to remove the terminal part of the phalanx, as if this was not done there was great likelihood of a recurrence.—Mr. LACY FIRTH said in his experience it was not necessary to remove more than the tumour itself and the cartilage surrounding it.

J. LACY FIRTH.

H. F. MOLE (*Hon. Sec.*).

